# Chemical profiling, antimicrobial and insecticidal evaluations of *Polygonum hydropiper* L

**DOI:** 10.1186/s12906-016-1491-4

**Published:** 2016-12-05

**Authors:** Muhammad Ayaz, Muhammad Junaid, Farhat Ullah, Abdul Sadiq, Muhammad Ovais, Waqar Ahmad, Sajjad ahmad, Anwar Zeb

**Affiliations:** 1 Department of Pharmacy, University of Malakand, Khyber Pakhtoonkhwa (KPK), 18000 Pakistan; 2 Cancer Biology Lab (MOSEL), Department of Biotechnology, Quaid-i-Azam University, Islamabad, Pakistan

**Keywords:** *Tribolium castaneum*, *Rhyzopertha dominica*, *Monomorium pharaonis*, *Anobium punctatum*, Larvicidal, Gas chromatography, Fungicidal and antibacterial activity

## Abstract

**Background:**

The emergence of multidrug resistant (MDR) pathogens is of great concern to the global health community. Our ability to effectively treat diseases is based on the discovery of potent drugs for the treatment of these challenging diseases. Traditional medicines are one of the major sources for the discovery of safe, effective and economical drug candidates. In order to validate its antibacterial, antifungal and insecticidal potentials with respect to traditional uses, we have screened for the first time *Polygonum hydropiper* against pathogenic bacterial, fungal strains and a variety of insects.

**Methods:**

*Polygonum hydropiper* samples including crude extract (Ph.Cr), subsequent fractions; *n*-hexane (Ph.Hex), chloroform (Ph.Chf), ethyl acetate (Ph.EtAc), *n*-Butanol (Ph.Bt), aqueous (Ph.Aq) and crude saponins (Ph.Sp) were tested against pathogenic bacterial and fungal strains. Insecticidal activities were performed against *Tribolium castaneum* and *Rhyzopertha dominica* and *Monomorium pharaonis.* Ph.Cr was analyzed by gas chromatography–mass spectrometry (GC-MS) for preliminary identification of chemical constituents.

**Results:**

In disc diffusion assay, Ph.Chf, Ph.Hex, Ph.EtAc and Ph.Sp exhibited highest activity against *Enterococcus faecalis.* MICs of Ph.Chf against *Enterococcus faecalis, Klebsiella pneumoniae, Escherichia coli, P. mirabilis, Staphylococcus aureus, Salmonella typhi* and *Pseudomonas aeruginosa* were 32.00, 13.33, 10.66, 5.33, 64.00, 8.66 and 10.66 μg/ml respectively. MFC’s of Ph.Chf against *Aspergillus fumigatus, Aspergillus flavus, Aspergillus niger* and *Fusarium oxysporum* were 16.66, 23.33, 125.00 and 46.66 μg/ml respectively. Ph.EtAc, Ph.Sp, Ph.Chf and Ph.Bt were most active fractions against *T. castaneum* and *R. dominica*. Ph.Sp being most active against *A. punctatum* exhibited LC_50_ of < 0.01 mg/ml. In GC-MS analysis of Ph.Cr, 124 compounds were identified among which several bioactive antibacterial, antifungal and insecticidal compounds were found.

**Conclusions:**

*P. hydropiper* samples exhibited broad spectrum of activity against bacterial and fungal strains. Our results support previously reported insecticidal properties of saponins and may provide scientific justification for the ethno-medicinal uses of the plant.

**Electronic supplementary material:**

The online version of this article (doi:10.1186/s12906-016-1491-4) contains supplementary material, which is available to authorized users.

## Background

The emergence of multidrug resistant (MDR) pathogens and life-threatening infections caused by these microorganisms is a global challenge for scientific community and some scientists speculate that we are going back to the pre-antibiotic era [1, 2]. The prevalence of microbial infections due to opportunistic pathogens, frequently characterized by high mortality rates, has increased during the past two decades [3]. Majority of antibiotics, currently applied in therapy, belong to drug classes discovered prior to 1970 [4]. The current expansion of antibacterial and antifungal drugs research has occurred since there is persistent need for developing new compounds to fight life-threatening infections [5]. Besides bacterial infections, fungi are the major causes of liver, lungs, mouth, blood and skin infections [6]. Systemic mycoses are most frequently caused by *Candida* genus yeasts and mould particularly, the *Aspergillus* genus. Moreover, many of the existing drugs are toxic, ineffective and enable infection recurrence because of being bacteriostatic/fungistatic in nature. Medicinal plants are potential sources of potent antimicrobial drugs and are used in many countries to treat infectious diseases [7]. Over the years, traditional phytotherapy is in practice for the treatment of microbial and non-microbial origin diseases [8]. World Health Organization (WHO) estimates that approximately 80% population of underdeveloped countries rely on medicinal plants for their primary health care [9].

Globally, researchers are trying to increase food production to fulfill the excessive food demand due to growing population. Unfortunately, insects are major contributors to extensive qualitative and quantitative loss of food grains, their products, and economically important crops. A total of 10–40% loss of food grains has been estimated due to insects globally. In spite of improved storage structures and traditional control practices, 70–90% of food grain cannot be stored for more than 6–12 months at farmer’s level [10]. Consequently, there is an immense need to employ safe insecticidal drugs and repellents to protect food grains from damages. In this regard, synthetic insecticidal agents are useful but their uses are limited due to development of insects’ resistance, high cost and deposition of toxic residue on grains. Therefore, there is a dire need to develop economical, safe, environment friendly and more effective insecticidal agents.

Approximately, fifteen hundred insecticidal plants has been reported among which ryania, nicotine, rotenone, sabadilla, pyrethrin and azadirachtin are commercially available [11]. *T. castaneum* (flour beetle) and *Rhyzopertha dominica* (grain borer) are common insect pests for food processing facilities such as mills, processing plants, warehouses and retail stores [12]. Both these insects have a long association with human stored foods and are commonly found in grain, cereal products, flour, peas, beans, nuts, dried fruits and spices [13]. *Anobium punctatum*, commonly known as woodworm or furniture beetle, is a common cause of damage to timber worldwide. During the last five decades, insecticidal treatments are extensively employed to minimize the insects’ risk especially for the timbers in buildings [14]. Besides this, *Monomorium pharaonis* is the main cause of damage to food stuff, store grains and wood products.

*P. hydropiper* is traditionally used to treat inflammation, gastrointestinal disturbances, neurological disorders and diarrhea [15]. Plant decoctions are used to treat an extensive range of ailments like dyspepsia, diarrhea, menorrhagia, hemorrhoids and skin itching [16]. Recently, *P. hydropiper* has been reported for anticholinesterase, antioxidant, phytotoxic, anthelmintic and anti-cancer potentials [17–20]. The current study was aimed to uncover the antibacterial, antifungal and insecticidal potentials of *P. hydropiper* extracts and sponins.

## Methods

### Plant collection, extraction and fractionation

*P. hydropiper* aerial parts (stem, leaves and flowers) were collected from Talash Valley, Khyber Pakhtoonkhwa, Pakistan in July, 2013. The plant was identified by Dr. Gul Rahim, botanical taxonomist. A sample was deposited at the herbarium, University of Malakand Chakdara (Dir), Pakistan with voucher no (H.UOM.BG.107). Plant material was cleansed, shade dried for 15 days and coarsely crushed with a cutter mill. Crude powder (4.5 kg) was soaked in 22 L of 80% methanol for 10 days with frequent shaking. This extraction with methanol was three times followed by filtration from muslin cloth [21]. The filtrate was concentrated using rotary evaporator (Heidolph Laborota 4000, Schwabach, Germany) under reduced pressure at 40 ^o^C, which resulted in 290 g (6.44%) of dark brown colored crude extract [22]. Ph.Cr (250 g) was processed for fractionation purpose following procedure we reported previously [17].

### Extraction of crude saponins

Saponins were extracted from 60 g of powdered plant material following our previously reported procedure [17]. Finally, 9 g of saponins with a percent yield of 15% were obtained.

### Gas chromatography–mass spectrometry (GC/MS) analysis

Ph.Cr was analyzed by means of an Agilent USB-393752 gas chromatograph (Agilent Technologies, Palo Alto, CA, USA) with HHP-5MS 5% phenylmethylsiloxane capillary column (30 m × 0.25 mm × 0.25 μm film thickness Restek, Bellefonte, PA) equipped with an flame ionization (FID) detector. Helium was used as carrier gas at a flow rate of 1 ml/min, and diluted samples (1/1000 in *n*-pentane, v/v) of 1.0 μl were injected manually in the splitless mode. GC/MS analysis of Ph.Cr was processed using an Agilent USB-393752 gas chromatograph (Agilent Technologies, Palo Alto, CA, USA) with a HHP-5MS 5% phenylmethylsiloxane capillary column (30 m × 0.25 mm × 0.25 μm film thickness Restek, Bellefonte, PA) outfitted with an Agilent HP-5973 mass selective detector in the electron impact mode (Ionization energy: 70 eV) working under the same experimental conditions as described for GC [23].

### Chemical and drugs

Nutrient agar (Oxoid Ltd, UK), Nutrient broth (Oxoid), Sabouraud’s dextrose agar (SDA), Dimethyl-Sulfoxide DMSO (Labscan Patumwan Bankok 10330 Thialand), Permethrin (CAS 52645-53-1) Sigma aldrich laborchemikalie GmbH, ceftriaxone (Geltis, Shaigan Pharmaceuticals), antibiotic discs (Oxoid) of ciprofloxacin, moxifloxacin, amoxicillin and gentamicin, amphotericin-B were used in the study. Solvents used were of analytical grade and were purchased from authorized dealer of Sigma Aldrich CHEMIE GmbH USA, Pakistan.

### Collection and identification of bacteria

Bacterial strains including *Staphylococcus aureus* (29213), *Enterococcus faecalis* (29212)*, Klebsiella pneumoniae* (700603)*, Escherichia coli* (739)*, Proteus mirabilis* (13315)*, Salmonella typhi* and *Pseudomonas aeruginosa* (27853) were used in the study. Bacterial strains were provided by Department of Microbiology, Quaid-i-Azam University Islamabad Pakistan. These strains were identified by different biochemical tests and were preserved in freeze-dried condition at 4 °C in stab slant agar until later use [24].

### Standardization of bacterial suspension

Bacterial cultures were grown for 24 h at 37 °C and suspension with cell density of 1 × 10^8^ CFU/ml, were prepared using McFarland standard and were further diluted to a cell density of 1 × 10^6^ CFU/ml using a UV visible spectrophotometer (Thermo electron corporation USA) at 625 nm. The standardization was maintained for the whole period of the study.

### Antibacterial investigations

#### Bacterial susceptibility pattern

Susceptibility pattern of selected bacterial strains was determined by disc diffusion method using standard antibiotic discs of ceftriaxone, ciprofloxacin, moxifloxacin, amoxicillin and gentamicin. Diameter of inhibitory zones indicated sensitivity or resistance to these antibiotics.

#### Disc diffusion assay

For determination of antibacterial potential of plant extracts, a qualitative to semi quantitative disc method was used following previously reported procedure [5]. Briefly, nutrient agar plates, prepared aseptically, were inoculated with test organisms under laminar flow hood. Sterile paper discs of 6 mm diameter (Whatman International, CAT: 2017-006) impregnated with different concentrations (25, 50, 100 μg/ml) of extracts were placed equidistantly onto the surface of the already inoculated Petri dishes using sterile forceps. Blank discs impregnated with DMSO/solvents were used as negative control whereas, ceftriaxone discs (25, 50, 100 μg/ml) were used as positive control. The plates were incubated at 37 °C for 24 h and zone of inhibition was measured around the discs.

#### Determination of Minimum Inhibitory Concentrations (MICs)

For determination of MICs, both broth and agar dilution methods approved by clinical and laboratory standard institute (CLSI) were used [25, 26]. For these tests, plant extracts in serial dilutions of 2-512 μg/ml were added to sterilized tube containing nutrient broth, so that the final concentration of the test samples were 2-512 μg/ml. Tubes were inoculated with the test microbes. Tubes were incubated using shaker incubator at 37 °C for 24 h.

#### Antifungal investigations

##### Fungal strains

Four fungal strains including *A. fumigatus*, *A. niger*, *A. flavus* and *F. oxysporum* were used to determine antifungal potential of plant extracts. Fungal strains were kindly provided by Department of Microbiology, Quaid-i- Azam University Islamabad Pakistan.

##### Preliminary antifungal activity

Before proceeding to detail antifungal studies, antifungal potential of all samples were performed. Briefly, each plant sample was prepared at concentration of 10 mg/ml and one ml was added to 9 ml SDA, already prepared in test tubes. These test tubes were inoculated with the fungal strains and were incubated at 27 °C for 7 days. Finally, test tubes were checked for inhibition of fungal growth [27].

##### Disc diffusion assay

Antifungal potentials of *P. hydropiper* extracts and spaonins were investigated by disc diffusion method as previously reported [28, 29]. Sabouraud dextrose Agar (SDA) plates were prepared and inoculated with the test fungi under laminar flow hood. Sterile paper discs of 6 mm diameter (Whatman International, CAT: 2017-006), impregnated with different concentrations of extracts and standard drug (125, 250 and 500 μg/ml) were placed equidistantly onto the surface of these Petri dishes and were incubated at 27 °C for 72 h. Diameter of Inhibitory zone around the discs was measured and was compared with standard drug.

##### Minimum Fungicidal Concentration (MFCs)

Minimum fungicidal concentrations (MFCs) of plant extracts were determined using agar dilution techniques in Sabouraud’s dextrose agar (SDA) and nutrient broth. SDA and nutrient broth (Oxoid Ltd, England) were prepared according to manufacturer specifications and serial dilutions of samples 2.5–1000 μg/ml were aseptically added to these tubes at 40 °C. The tubes were inoculated by adding one loopful of already prepared fungal suspensions and were incubated at 27 °C. After 7–10 days, tubes were observed for fungal growth and MFCs were considered the lowest concentration which inhibited fungal growth [27].

##### Collection and identification of the insects

The *Tribolium castaneum* (flour beetle) was collected from the flour mill Chakdara in the proximity of University of Malakand. Similarly, the *Rhyzopertha dominica* (grain borer) was collected from the grocery shop in university town, University of Malakand. *Anobium punctatum* (wood worm) was collected from timber market Chakdara. Likewise, the *Monomorium pharaonis* (Pharaoh ants) were collected from the main campus, University of Malakand. All the insects were identified and authenticated by Saeed Ahmad, Assistant Professor, Department of Zoology, University of Malakand.

##### Insecticidal activity against *T. castaneum* and *R. dominica*

Insecticidal potential of plant extracts was tested on adult insects of *T. castaneum* and *R. dominica*, using previously reported procedure [30]*.* Different concentrations (125–500 μg/ml) of plant extracts were prepared in methanol. Filter papers were dipped in these solutions and were transferred to sterile Petri dishes. The plates were left overnight for evaporation of the solvent. Thirty healthy and active insects of both species were transferred to test group Petri dishes, positive control (Permethrin) and negative control groups Petri dishes and were kept in growth chamber at 27 °C for 24 h with 50% relative humidity. Percent insecticidal activity was determined from the number of dead insects after 24 and 48 h.

##### Anti-anobium investigations

The anti-anobium activity of Plant samples was evaluated following previously reported procedure [31]. Using this procedure, different plant extracts were assayed for lethality against *A. punctatum*. Briefly, different concentrations of samples were prepared by dissolving 100 mg/ml in respective solvents and were further diluted. Sterilized filter papers were put in sterile Petri dishes. Solvents were transferred to Petri dishes and kept overnight for the removal of solvents. *A. punctatum,* 25 larva’s were transferred to each Petri dish and were kept at room temperature for 24 h. The number of dead and alive larva were counted

##### Anti-pharaoh investigations

Anti-Pharaoh potential of samples were determined by contact toxicity method, following previously reported procedure [32]. Sample solutions in concentrations of 12.5–50 mg/ml were added to sterile Petri dishes containing filter paper and were left overnight for evaporation of solvents. Thereafter, 30 pharaohs were transferred to each Petri dish and were incubated at room temperature for 24 h. Finally, the numbers of dead and alive Pharaoh were counted in each Petri dish. The Petri dishes containing filter paper plus distilled water served as a control.

##### Estimation of LC_50_ values

Median lethal concentrations (LC_50_) were calculated for insecticidal, anti-Anobium and anti-Pharaoh activities, using Microsoft Excel program.

##### Statistical analysis

All the experiments were performed in triplicate and values were expressed as means ± SEM. One way ANOVA followed by multiple comparison Dunnett’s test was used for the comparison of positive control with the test groups. The *P* values less than 0.05 were considered as statistically significant.

## Results

### Antibacterial activity

#### Bacterial susceptibility pattern

**Fig. 1:**
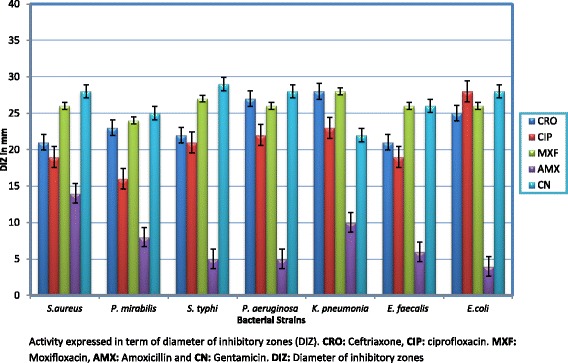
Susceptibility pattern of bacterial strains exposed to standard antibiotic discs

Majority of bacterial strains were susceptible to the tested antibiotics except amoxicillin to whom bacterial strains were resistant (Fig. 1).

#### Disc diffusion assay

In disc diffusion assay Ph.Chf, Ph.Hex, Ph.EtAc and Ph.Sp were found most active against *E. faecalis* as shown in Table 1. Most of fractions were less active against *K. pneumonia* whereas, Ph.Chf, Ph.Hex and Ph.Cr were moderately effective. Ph.Aq and Ph.Sp were most active against *E. coli* with inhibitory zones of 26.66 ± 1.20 and 23.00 ± 0.00 mm respectively. Furthermore, Ph.Cr, Ph.Bt, Ph.EtAc and Ph.Chf were active against *P. mirabilis* in comparison to other fractions. Ph.Hex, Ph.Bt and Ph.Sp were most active against *S. aureus* with inhibitory zones of 26.33 ± 0.88, 23.00 ± 1.15 and 22.00 ± 1.52 mm respectively. Ph.Chf was most active against S. *typhi* and *P. aeruginosa.*

**Table 1:** Antibacterial activity of *Polygonum hydropiper* extracts and saponins against bacterial strains

Samples	Conc. μg/ml	Diameter of the inhibitory zone (mm) Mean ± SEM (*n* = 3)						
		*Enterococcus faecalis*	*Klebsiella pneumonia*	*Escherichia coli*	*Proteus mirabilis*	*Staphylococcus aureus*	*Salmonella typhi*	*Pseudomonas aeruginosa*
Crude (Ph.Cr)	25	5.33 ± 0.88	6.33 ± 0.66	0.00 ± 0.00	11.00 ± 0.00	10.00 ± 0.57	11.33 ± 1.20	15.66 ± 1.85
	50	14.00 ± 0.57	10.33 ± 0.33	0.00 ± 0.00	17.33 ± 0.88	14.33 ± 0.33	14.00 ± 0.57	20.33 ± 0.88
	100	17.33 ± 0.88	14.00 ± 0.00	9.00 ± 0.57	34.00 ± 1.15	18.66 ± 0.66	21.33 ± 0.88	25.33 ± 1.45
Ethyl acetate (Ph.EtAc	25	9.33 ± 0.33	0.00 ± 0.00	0.00 ± 0.00	11.66 ± 1.20	8.33 ± 0.88	8.00 ± 0.57	13.00 ± 0.57
	50	18.33 ± 0.88	7.00 ± 0.57	8.33 ± 0.88	18.33 ± 0.66	13.66 ± 1.20	15.66 ± 0.66	18.33 ± 0.66
	100	27.00 ± 0.57	13.33 ± 0.88	15.00 ± 0.00	27.66 ± 2.18	18.33 ± 0.88	20.73 ± 1.00	24.00 ± 0.57
Chloroform (Ph.Chf)	25	13.66 ± 0.33	0.00 ± 0.00	8.00 ± 0.57	12.66 ± 3.40	7.33 ± 0.88	17.33 ± 0.66	18.66 ± 1.20
	50	19.00 ± 0.57	8.00 ± 0.57	14.33 ± 0.33	15.33 ± 2.02	11.66 ± 1.45	23.00 ± 0.57	23.00 ± 1.00
	100	31.66 ± 1.20	15.60 ± 1.15	18.00 ± 1.52	21.00 ± 1.73	16.00 ± 1.15	29.33 ± 0.88	31.66 ± 2.18
Butanol (Ph.Bt)	25	7.00 ± 1.15	0.00 ± 0.00	0.00 ± 0.00	14.00 ± 0.57	13.66 ± 1.20	11.33 ± 0.66	7.33 ± 0.88
	50	11.00 ± 1.15	0.00 ± 0.00	0.00 ± 0.00	19.66 ± 1.20	18.66 ± 1.45	14.00 ± 1.52	12.66 ± 0.66
	100	21.00 ± 0.00	8.00 ± 0.00	11.33 ± 0.88	29.00 ± 0.00	23.00 ± 1.15	19.00 ± 0.57	16.00 ± 1.52
n-Hexane (Ph.Hex)	25	11.66 ± 0.66	0.00 ± 0.00	0.00 ± 0.00	0.00 ± 0.00	11.00 ± 1.15	8.66 ± 1.66	12.66 ± 0.33
	50	17.66 ± 1.66	9.66 ± 0.33	7.66 ± 1.20	9.33 ± 0.66	17.33 ± 2.18	14.00 ± 1.00	17.00 ± 0.57
	100	30.00 ± 0.57	15.00 ± 0.57	12.00 ± 0.57	14.00 ± 0.57	26.33 ± 0.88	20.33 ± 0.88	21.00 ± 0.00
Saponins (Ph.Sp)	25	6.60 ± 1.15	0.00 ± 0.00	11.00 ± 1.00	0.00 ± 0.00	10.66 ± 2.02	22.00 ± 0.57	15.00 ± 0.00
	50	10.33 ± 1.45	00.00 ± 0.00	16.00 ± 0.57	0.00 ± 0.00	15.00 ± 0.57	26.33 ± 0.33	22. 33 ± 1.20
	100	26.66 ± 1.20	7.33 ± 0.88	23.00 ± 0.00	8.66 ± 1.20	22.00 ± 1.52	30.66 ± 1.20	27.00 ± 0.57
Aqueous (Ph.Aq)	25	4.00 ± 0.00	0.00 ± 0.00	6.00 ± 1.15	0.00 ± 0.00	0.00 ± 0.00	0.00 ± 0.00	6.33 ± 0.66
	50	13.00 ± 0.57	0.00 ± 0.00	10.33 ± 0.33	0.00 ± 0.00	7.00 ± 0.57	10.33 ± 0.66	10.88 ± 0.33
	100	20.33 ± 0.33	10.33 ± 1.45	26.66 ± 1.20	11.33 ± 1.45	14.33 ± 2.02	15.60 ± 1.15	13.00 ± 0.66
Positive Control	25	19.33 ± 2.02	24.00 ± 0.57	23.33 ± 1.45	22.57 ± 0.57	16.66 v 0.88	20.33 ± 0.88	22.33 ± 0.66
	50	25.00 ± 0.57	29.33 ± 0.66	29.66 ± 1.20	27.66 ± 0.88	23.33 ± 1.52	27.00 ± 0.57	29.00 ± 0.57
	100	31.66 ± 1.45	35.33 ± 0.88	34.66 ± 0.88	31.00 ± 1.52	27.00 ± 1.15	34.66 ± 1.20	36.66 ± 1.20

Results expressed as diameter of inhibitory zones (DIZ). Each value represent Mean ± SEM of three independent experimental results. Positive Control: Ceftriaxone

#### Minimum Inhibitory Concentrations (MICs)

Results of MICs are summarized in Table 2. Ph.Chf and Ph.Sp were most active against bacterial strains. The MICs of Ph.Chf against *E. faecalis, K. pneumonia, E. coli, P. mirabilis, S. aureus, S. typhi* and *P. aeruginosa* were 32.00 ± 0.00, 13.33 ± 2.66, 10.66 ± 2.66, 5.33 ± 1.33, 64.00 ± 0.00, 8.66 ± 0.66 and 10.66 ± 2.66 μg/ml respectively. Ph.Sp has exhibited lower MIC values against *E. faecalis* (10.66 ± 2.66)*, K. pneumonia* (32.00 ± 0.00)*, E. coli* (26.66 ± 5.33)*, P. mirabilis* (6.66 ± 1.33)*, S. aureus* (128.00 ± 0.00)*, S. typhi* (53.33 ± 10.66) and *P. aeruginosa* (6.66 ± 1.33) μg/ml. Results of these fractions were comparable to positive control.

**Table 2:** Minimum Inhibitory concentrations (MICs) of solvent extracts from *Polygonum hydropiper* against bacterial strains

Bacterial strains	Minimum inhibitory concentrations (MICs) in μg/ml.							
	Crude (Ph.Cr)	n-Hexane (Ph.Hex)	Ethyl acetate (Ph.EtAc)	Butanol (Ph.Bt)	Chloroform (Ph.Chf)	Aqueous (Ph.Aq)	Saponins (Ph.Sp)	Ceftriaxone
*Enterococcus faecalis*	128.00 ± 0.00	64.00 ± 0.00	26.66 ± 5.33	21.33 ± 5.33	32.00 ± 0.00	>512	10.66 ± 2.66	8.00 ± 0.00
*Klebsiella pneumoniae*	53.33 ± 10.66	42.66 ± 10.66	13.33 ± 2.66	26.66 ± 5.33	13.33 ± 2.66	256.00 ± 0.00	32.00 ± 0.00	4.00 ± 0.00
*Escherichia coli*	64.00 ± 0.00	128.00 ± 0.00	64.00 ± 0.00	128.00 ± 0.00	10.66 ± 2.66	> 512	26.66 ± 5.33	4.00 ± 0.00
*Proteus mirabilis*	21.33 ± 5.33	256.00 ± 0.00	32.00 ± 0.00	128.00 ± 0.00	5.33 ± 1.33	128.00 ± 0.00	6.66 ± 1.33	8.00 ± 0.00
*Staphylococcus aureus*	512.00 ± 0.00	128.00 ± 0.00	512.00 ± 0.00	>512	64.00 ± 0.00	256.00 ± 0.00	128.00 ± 0.00	16.00 ± 0.00
*Salmonella typhi*	64.00 ± 0.00	128.00 ± 0.00	64.00 ± 0.00	13.33 ± 2.66	8.66 ± 0.66	128.00 ± 0.00	53.33 ± 10.66	16.00 ± 0.00
*Pseudomonas aeruginosa*	26.66 ± 5.33	32.00 ± 0.00	64.00 ± 0.00	32.00 ± 0.00	10.66 ± 2.66	512.00 ± 0.00	6.66 ± 1.33	4.00 ± 0.00

MIC were determined at concentrations range of 2, 4, 8, 16, 32, 64, 128, 256 and 512 μg/ml. Each value represent Mean ± SEM of three independent experimental readings

#### Antifungal activity

##### Preliminary antifungal assay

Results were expressed in the form of complete growth inhibition (+++), moderate inhibition (++) and partial (+) inhibition. All fractions showed antifungal activities. Ph.Chf was found most effective, causing complete inhibition of visible fungal growth against all tested strains (Table 3). Among other fractions, Ph.Sp, Ph.Cr and Ph.Bt showed prominent antifungal activity. Antifungal action of other fractions was mild to moderate.

**Table 3:** Preliminary antifungal activity of *P. hydropiper* extracts and crude saponins

Fungal strains	Inhibition of fungal growth							
	Crude (Ph.Cr)	n-Hexane (Ph.Hex)	Ethyl acetate (Ph.EtAc)	Butanol (Ph.Bt)	Chloroform (Ph.Chf)	Aqueous (Ph.Aq)	Saponins (Ph.Sp)	Positive Control
*Aspergillus fumigatus*	+++	++	+++	+++	+++	++	+++	+++
*Aspergillus flavus*	+++	++	++	++	+++	+	+++	+++
*Aspergillus niger*	++	++	+++	++	+++	-	++	+++
*Fusarium oxysporum*	++	+++	+++	++	+++	-	+++	+++

Antifungal action expressed as complete inhibition (+++), Medium inhibition (++), Mild inhibition (+) and no inhibition (-) of fungal growth. Positive Control: Amphotericin-B

##### Antifungal disc diffusion assay

Results of antifugal activity are given in Table 4. Ph.Chf, Ph.Bt and Ph.EtAc were most active against *A. fumigatus* scoring inhibitory zones of 26.00 ± 1.73, 22.33 ± 1.45 and 21.33 ± 0.88 mm respectively at 500 μg/ml. Ph.Chf and Ph.Aq exhibited inhibitory zones of 22.66 ± 1.20 and 24.33 ± 1.45 mm respectively against *A. flavus* at highest tested concentration. Further, Ph.Bt, Ph.Chf and Ph.Aq showed highest activity against *A. niger*. In activity against *F. oxysporum*, Ph.Bt and Ph.Aq were found more effective in comparison to other fractions. Rest of the fractions showed moderate activity against the fungal strains.

**Table 4:** Antifungal activity of *P. hydropiper* extracts against fungal strains in disc diffusion assay

Samples/Fractions	Fungal strains (DIZ in mm *n* = 3 SEM)				
	Conc. μg/ml	*Aspergillus fumigatus*	*Aspergillus flavus*	*Aspergillus niger*	*Fusarium oxysporum*
Crude (Ph.Cr)	125	6.33 ± 0.33	9.00 ± 1.15	5.00 ± 0.00	9.66 ± 1.76
	250	10.00 ± 0.57	16.00 ± 0.57	8.50 ± 1.00	13.33 ± 0.88
	500	16.00 ± 0.00	20.33 ± 0.88	12.00 ± 0.57	18.00 ± 0.00
n-Hexane (Ph.Hex)	125	6.66 ± 0.66	8.00 ± 0.57	8.00 ± 1.15	4.00 ± 0.50
	250	9.33 ± 0.88	11.00 ± 1.15	11.33 ± 0.88	7.30 ± 1.50
	500	11.00 ± 1.15	16.33 ± 0.33	16.00 ± 1.00	10.00 ± 1.00
Ethyl acetate (Ph.EtAc)	125	14.00 ± 0.57	7.00 ± 1.15	13.66 ± 0.66	6.33 ± 1.45
	250	16.00 ± 0.33	9.33 ± 0.88	17.33 ± 2.02	9.00 ± 1.15
	500	21.33 ± 0.88	17.00 ± 0.00	19.66 ± 1.76	15.00 ± 0.00
Butanol (Ph.Bt)	125	13.00 ± 0.50	9.00 ± 0.50	15.00 ± 0.00	12.33 ± 0.66
	250	18.00 ± 1.70	14.00 ± 1.73	21.66 ± 1.45	18. 00 ± 0.50
	500	22.33 ± 1.45	17.33 ± 1.45	26.00 ± 2.30	26.00 ± 1.73
Chloroform (Ph.Chf)	125	13.00 ± 1.15	13.00 ± 1.15	14.33 ± 0.88	9.33 ± 2.02
	250	20.33 ± 0.88	17.00 ± 2.30	19.00 ± 1.45	13.00 ± 0.57
	500	26.00 ± 1.73	22.66 ± 1.20	23.00 ± 1.15	16.00 ± 2.30
Aqueous (Ph.Aq)	125	10.33 ± 0.88	15.00 ± 1.15	12.00 ± 00	12.30 ± 0.33
	250	13.00 ± 1.15	18.00 ± 1.73	17.00 ± 1.45	17.00 ± 1.73
	500	17.00 ± 2.30	24.33 ± 1.45	21.00 ± 0.57	28.66 ± 0.88
Saponins (Ph.Sp)	125	8.33 ± 0.88	11.00 ± 0.00	7.00 ± 0.00	9.00 ± 2.30
	250	11.66 ± 0.66	16.00 ± 0.50	12.33 ± 1.20	12.66 ± 1.20
	500	16.00 ± 0.57	18.33 ± 1.15	15.00 ± 1.15	15.00 ± 0.00
Positive Control	125	19.33 ± 0.88	23.66 ± 1.76	17.00 ± 2.88	22.66 ± 1.20
	250	26.00 ± 1.15	30.66 ± 2.33	22.00 ± 2.00	28.66 ± 2.60
	500	33.00 ± 0.57	36.00 ± 1.73	29.00 ± 1.15	35.33 ± 2.02
N. Control	----	-----	----	----	----

Positive Control: Amphotericin-B. Results are expressed as diameter of inhibitory zone (DIZ). Each value represent Mean ± SEM of three independent experimental readings

##### Minimum Fungicidal Concentrations (MFCs)

In MFCs determination assay, Ph.Chf was highly effective against *A. fumigatus, A. flavus, A. niger* and *F. oxysporum* exhbiting MFCs of 16.66 ± 3.33, 23.33 ± 8.81, 125.00 ± 0.00 and 46.66 ± 6.66 μg/ml respectively (Table 5). Ph.Sp, Ph.EtAc and Ph.Bt and were most effective against *A. fumigatus* showed MFCs of 20.00 ± 0.00, 16.66 ± 3.33 and 33.33 ± 6.66 μg/ml. Ph.Aq was least effective against the tested fungi exhibiting MFCs of > 1000 μg/ml against *A. flavus* and *F. oxysporum*.

**Table 5:** Minimum fungicidal concentrations (MFCs) of *Polygonum hydropiper* extracts and saponins

Fungal strains	Minimum Fungicidal concentrations (MFCs) in μg/ml							
	Crude (Ph.Cr)	n-Hexane (Ph.Hex)	Ethyl acetate (Ph.EtAc)	Butanol (Ph.Bt)	Chloroform (Ph.Chf)	Aqueous (Ph.Aq)	Saponins (Ph.Sp)	Positive Control
*Aspergillus fumigatus*	73.33 ± 6.66	116.66 ± 8.33	16.66 ± 3.33	33.33 ± 6.66	16.66 ± 3.33	500.00 ± 0.00	20.00 ± 0.00	8.33 ± 1.66
*Aspergillus flavus*	250.00 ± 0.00	73.33 ± 6.66	60.00 ± 0.00	125.00 ± 0.00	23.33 ± 8.81	> 1000	53.33 ± 6.66	6.66 ± 1.66
*Aspergillus niger*	100.00 ± 0.00	> 1000	40.00 ± 0.00	93.33 ± 6.66	125.00 ± 0.0	500.00 ± 0.00	33.33 ± 6.66	16.66 ± 3.33
*Fusarium oxysporum*	86.66 ± 6.66	500.00 ± 0.00	93.33 ± 6.66	250.00 ± 0.00	46.66 ± 6.66	>1000	40.00 ± 0.00	20.00 ± 0.00

Minimum Fungicidal concentrations (MFCs) were measured at concentration range of 2.5–512 μg/ml after serial dilutions. Experiments were performed in triplicate. Positive Control: Amphotericin-B

##### Insecticidal study against *T. castaneum* and *R. dominica*

Results of insecticidal activity against *T. castaneum* and *R. dominica* are given in Table 6. Among different fractions, Ph.EtAc, Ph.Sp, Ph.Chf and Ph.Bt were most active exhibiting LC_50_ of 20, 110, 80 and 130 μg/ml respectively*.* Moreover, insecticidal action of Ph.EtAc, Ph.Sp, Ph.Chf and Ph.Cr were most prominent against *R. dominica* with LC_50_ of 57, 28, 110 and 25 μg/ml respectively.

**Table 6:** Insecticidal activity of solvent extracts from *Polygonum hydropiper* against *Tribolium castaneum* and *Rhyzopertha dominica*

Samples/Fractions	Total Insects	Conc. (μg/ml)	*Tribolium castraneum* killed	Percent mortality	LC_50_ μg/ml	*Rhyzopertha dominica* Killed	Percent mortality	LC_50_ μg/ml
Crude (Ph.Cr)	30	125	12.00 ± 0.57	40.00^***^	255	19.00 ± 0.00	63.33**	25
	30	250	14.33 ± 0.66	47.76^***^		21.00 ± 1.15	70.00**	
	30	500	18.33 ± 0.33	61.10^**^		22. 67 ± 0.88	75.56**	
n-Hexane (Ph.Hex)	30	125	12.66 ± 0.33	42.20^***^	360	13.00 ± 0.57	43.33***	190
	30	250	15.66 ± 0.66	62.20^**^		16.66 ± 0.33	55.53***	
	30	500	18.67 ± 0.33	62.23^**^		19.00 ± 0.58	63.33**	
Ethyl acetate (Ph.EtAc)	30	125	17.66 ± 0.66	58.86^***^	80	15.33 ± 0.88	51.10***	110
	30	250	20.00 ± 0.57	66.66^**^		23.00 ± 1.15	76.66**	
	30	500	24.00 ± 0.57	80.00^*^		26.33 ± 0.33	87.76*	
Butanol (Ph.Bt)	30	125	21.33 ± 0.33	71.10^**^	20	19.66 ± 1.88	65.53**	57
	30	250	24.00 ± 0.57	80.00^*^		25.00 ± 1.15	83.33*	
	30	500	26.67 ± 0.89	88.90^*^		28.00 ± 0.00	93.33	
Chloroform (Ph.Chf)	30	125	4.66 ± 0.88	15.53^***^	>500	5.00 ± 0.57	16.55***	>500
	30	250	6.33 ± 0.33	21.10^***^		9.00 ± 1.15	30.00***	
	30	500	8.67 ± 0.89	28.90^***^		11. 67 ± 0.88	38.90***	
Aqueous (Ph.Aq)	30	125	14.00 ± 0.00	46.66^***^	130	9.66 ± 0.88	32.20***	300
	30	250	21.00 ± 1.15	70.00^**^		14.00 ± 0.57	46.66***	
	30	500	23.33 ± 1.21	77.77^**^		18.33 ± 0.33	61.10**	
(Saponins) Ph.Sp	30	125	15.33 ± 0.88	51.10^***^	110	21.33 ± 1.20	71.10**	28
	30	250	22.00 ± 0.00	73.33^**^		24.00 ± 0.00	80.00*	
	30	500	25.00 ± 0.00	83.33^*^		27.00 ± 0.00	90.00	
Positive Control	30	125	24.00 ± 0.00	80.00	15	24.66 ± 0.33	82.20	9
	30	250	27.66 ± 0.88	92.20		27.33 ± 0.88	91.10	
	30	500	30.00 ± 0.00	100.00		30.00 ± 0.00	100.00	
Negative Control	30	---	0 ± 0.00	0 ± 0.00	---	0 ± 0.00	0 ± 0.00	

Data was represented as mean ± SEM (*n* = 3) of three independent experimental readings. Positive control: Permethrin. Negative Control: solvents used for dissolution. Values significantly different when compared to standard drug *: 0.05, **: 0.01 and ***: 0.001 at 90% confidence interval

##### Anti- anobium activity

Results of larvicidal activity against *A. punctatum* are given in Table 7. Ph.Sp was found most active causing 94.64, 96.00 and 100.00% lethality of tested larvae at 12.5, 25 and 50 mg/ml respectively. Saponins activity was comparable with standard drug permethrin, causing 100% death of all larvae with LC_50_ of < 0.01. Among other fractions, Ph.Chf, Ph.EtAc and Ph.Cr were most active causing 93.32, 84.00 and 80.00% lethality against *A. punctatum* with LC_50_ 1.16, 6.35 and 0.93 mg/ml respectively at 50 mg/ml. Ph.Hex, Ph.Bt and PhAq showed moderate activity.

**Table 7:** Larvicaidal activity of *Polygonum hydropiper* extracts against *Anobium punctatum*

Samples	Dose (mg/ml)	Total treated	No. Repeated	Average lethality	%lethality mean ± SEM	LC_50_ (mg/ml)
Crude (Ph.Cr)	12.5	25	3	17.66 ± 0.66	70.64	0.93
	25	25		18.66 ± 0.33	74.64	
	50	25		20.00 ± 0.57	80.00	
n-Hexane (Ph.Hex)	12.5	25	3	8.66 ± 0.88	34.64	27.32
	25	25		12.33 ± 0.88	49.32	
	50	25		14.66 ± 1.20	58.64	
Chloroform (Ph.Chf)	12.5	25	3	19.33 ± 0.88	77.32	1.16
	25	25		22.33 ± 1.20	89.32	
	50	25		23.33 ± 0.66	93.32	
Ethyl acetate (Ph.EtAc)	12.5	25	3	15.66 ± 1.33	62.64	6.35
	25	25		17.66 ± 2.33	70.64	
	50	25		21.00 ± 1.15	84.00	
Aqueous (Ph.Aq)	12.5	25	3	9.66 ± 1.20	38.64	53.24
	25	25		11.00 ± 1.15	44.00	
	50	25		12.33 ± 0.88	49.32	
Butanol (Ph.Bt)	12.5	25	3	16.66 ± 0.33	66.64	2.28
	25	25		17.33 ± 0.88	69.32	
	50	25		19.66 ± 2.33	78.64	
(Saponins) Ph.Sp	12.5	25	3	23.66 ± 0.33	94.64	<0.01
	25	25		24.00 ± 0.00	96.00	
	50	25		25.00 ± 0.00	100.00	
Positive Control	12.5	25	3	25.00 ± 0.00	100.0	<0.01
	25	25		25.00 ± 0.00	100.0	
	50	25		25.00 ± 0.00	100.0	
Negative Control	---	25	3	0.00	0.00	0.00

Each value represent Mean ± SEM of three independent experimental readings. Results were expressed as % mortality and LC_50_ (mg/ml). Negative Control: Distilled Water, Positive Control: permethrin

##### Anti-pharaoh activity

In Anti-Pharaoh investigations Ph.Sp was found most active against the tested ants showing 93.30, 100.00 and 100.00% lethality at concentrations of 12.5, 25 and 50 mg/ml respectively with LC_50_ of < 0.01 mg/ml. Activity of Ph.Sp was comparable with standard drug at the same tested concentration. Ph.Chf was also equally effective, causing 83.30, 86.65 and 96.65% death of *M. pharaonis* at concentrations of 12.5, 25 and 50 mg/ml with LC_50_ of < 0.01 mg/ml. All other fractions showed mild to moderate activity as shown in Table 8.

**Table 8:** Anti-Pharaoh investigations of *P. hydropiper* extracts and saponins

Samples	Dose (mg/ml)	Total treated	No. Repeated	Average lethality	Percent lethality	LC_50_ (mg/ml)
Crude (Ph.Cr)	12.5	20	3	8.66 ± 0.66	43.30	33.54
	25	20		9.00 ± 1.15	45.00	
	50	20		12.66 ± 2.33	63.30	
n-Hexane (Ph.Hex)	12.5	20	3	5.00 ± 0.57	25.00	54.82
	25	20		7.66 ± 0.88	38.30	
	50	20		9.33 ± 0.88	46.65	
Chloroform (Ph.Chf)	12.5	20	3	16.66 ± 1.33	83.30	<0.01
	25	20		17.33 ± 0.66	86.65	
	50	20		19.33 ± 0.88	96.65	
Ethyl acetate (Ph.EtAc)	12.5	20	3	12.00 ± 0.00	60.00	5.91
	25	20		14.00 ± 1.15	70.00	
	50	20		15.66 ± 0.33	78.30	
Aqueous (Ph.Aq)	12.5	20	3	9.66 ± 1.20	48.30	17.17
	25	20		10.66 ± 1.33	53.30	
	50	20		12.33 ± 0.88	61.65	
Butanol (Ph.Bt)	12.5	20	3	12.00 ± 1.15	60.00	6.13
	25	20		13.66 ± 0.88	68.30	
	50	20		15.66 ± 0.66	78.30	
(Saponins) Ph.Sp	12.5	20	3	18.66 ± 0.33	93.30	<0.01
	25	20		20.00 ± 0.00	100.00	
	50	20		20.00 ± 0.00	100.00	
Positive Control	12.5	25	3	25.00 ± 0.00	100.0	<0.01
	25	25		25.00 ± 0.00	100.0	
	50	25		25.00 ± 0.00	100.0	
Negative Control	---	25	3	0.00	0.00	0.00

Negative Control: Distilled Water; Positive Control: Permethrin

##### GC-MS analysis

**Fig. 2:**
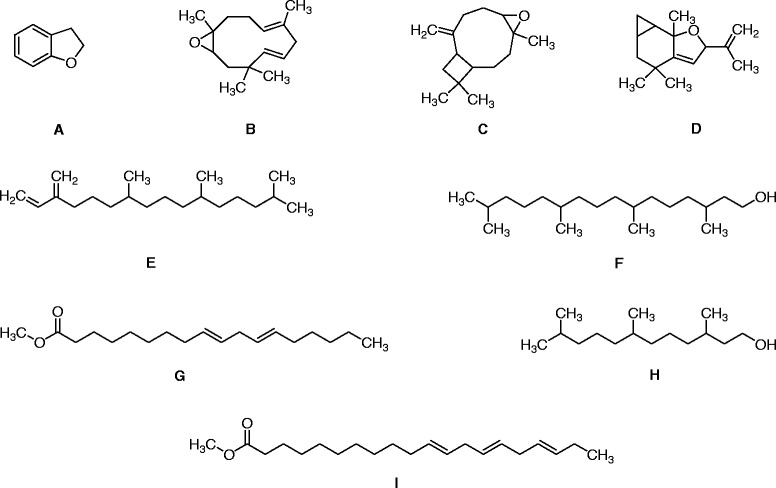
Major compounds identified in GC-MS analysis of methanolic extract from *P. hydropiper*

In GC-MS analysis of Ph.Cr, 124 compounds were identified (Additional file 1: Table S1). Overall, nine compounds were found dominant including 2,3-dihydro benzofuran, humulene oxide, caryophyllene epoxide, 2H-cyclopropa benzofuran, neophytadiene 7,11,15 trimethyl,3-methylene-1-hexadecene, 3,7,11,15-tetramethyl-2-hexadecen-1-ol, 3,7,11,15-Tetramethyl-2-hexadecen-1, 9,12-octadecadienoic acid methyl ester, (*E,E*)-methyl linolelaidate and 11,14,17-eicosatrienoic acid, methyl ester with concentrations of 7.89, 3.54, 3.68, 3.18, 25.2, 6.44, 10.71, 3.41 and 5.84% respectively (Fig. 2).

##### Identification of bioactive compounds

**Fig. 3:**
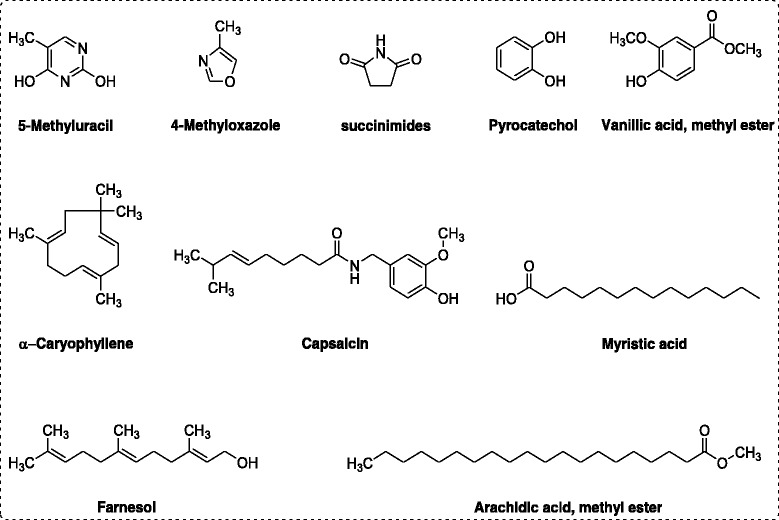
Bioactive compounds identified in GC-MS analysis of methanolic extract from *P. hydropiper*

Several bioactive antibacterial, antifungal and insecticidal compounds were identified in GC, GC-MS analysis of Ph.Cr (Fig. 3). These compounds include 4-methyloxazole, succinimide, pyrocatechol, caryophyllene, vanillic acid, farnesol, Myristic acid, arachidic acid methyl ester and capsaicin.

## Discussion

Infectious diseases are among the leading health problems, accounting for 41% of global disease Burdon [33]. The development of resistance by multidrug resistant (MDR) pathogens is a major problem in the chemotherapeutic management of infectious diseases. Due to the development of resistance against synthetic drugs, researchers are focusing on natural products to find novel antibacterial, antifungal and anti-parasitic agents [34–36]. Plant based remedies are rich sources of safe and effective drugs and are used throughout the history of mankind in crude form as well as in the form of pure isolated compounds [37]. A variety of medicinal plants have been indicated for the treatment of infectious diseases in different phytotherapy manuals due to their reduced toxicity, fewer side effects and frequent availability. Different studies have been conducted on the antimicrobial potentials of plants and their efficacy has been reported in the treatment of urinary tract infections (UTIs), respiratory, cutaneous infections, neurological and gastrointestinal disorders [38, 39].

In our current investigations, *P. hydropiper* extracts and saponins revealed a broad spectrum of activity against pathogenic bacterial and fungal strains. Antibacterial and antifungal potential of these fractions can be attributed to their phenolic contents and the presence of different pharmacologically active compounds. As indicated by their MICs and DIZ values, Ph.Chf, Ph.Hex and Ph.Sp showed most prominent activity against the tested bacterial strains whereas, Ph.Aq was least active. DIZ and MICs of these fractions correlated well with each other in relation to antibacterial activities. Broadly, samples were more active against Gram negative strains in comparison to Gram positive. In antifungal assays, Ph.EtAc, Ph.Bt, Ph.Chf and Ph.Sp exhibited prominent activity against fungal strains whereas, Ph.Aq was found least effective in MFCs assay (Tables 1, 2, 3, 4 and 5). Majority of fungal stains were inhibited at MFCs range of 16.66–1000 μg/ml.

Several bioactive compounds were identified in the GC-MS spectra of *P. hydropiper* including thymin, 4-methyloxazole, succinimide, vanillic acid, caryophyllene, farnesol, capsaicin, myristic acid, arachidic acid, methyl palmitate etc (Additional file 1: Table S1). Thymin, 4-methyloxazole, succinimide, pyrocatechol and caryophyllene has been previously reported for antibacterial and antifungal potential [40–45]. Furthermore, phenolic acid, farnesol, myristic acid, Arachidic acid methyl ester and capsaicin has been demonstrated against pathogenic bacteria and fungi [46–52]. The antimicrobial action of *P. hydropiper* can be attributed to the presence of these bioactive compounds.

Higher plants are good sources of novel compounds that can be used to develop environment friendly insecticidal drugs [53]. Insecticidal potentials of several plants against different insect pests has been reported by several groups [54]. A possibly interesting group of molecules is the saponins, a class of steroidal or triterpenoidal secondary plant metabolites having divergent biological activities [55]. *T. castaneum* and *R. dominica* are considered major pests of stored grains and food products. Annual post-harvest losses resulting from insect infestations, microbial deterioration and others factors is estimated to be 10–25% worldwide [56]. Control of these insects relies heavily on the utilization of synthetic insecticides and fumigants. However, their extensive use has led to some stern problems including development of insect strains resistant to insecticides, deposition of toxic residues on stored grain, toxicity to users and high costs of application [57]. There is critical need to develop safe and cost-effective alternatives which are convenient for user and environment friendly.

In our current insecticidal study, Ph.EtAc, Ph.Sp and Ph.Chf were most effective against *T. castaneum* with LC_50_ of 20, 110 and 80 μg/ml respectively which was comparable with standard drug permethrin. Similarly, Ph.EtAc, Ph.Sp and Ph.Chf were also most effective against *R. dominica* with LC_50_ of 57, 28 and 110 μg/ml respectively. Insecticidal potential of these fractions was comparable with the positive control at the same tested concentration. In larvicidal activity against *A. punctatum,* we observed that Ph.Sp were highly active with LC_50_ of < 0.01 mg/ml which was comparable with standard drug permethrin at the same tested concentration. Larvicidal potentials of Ph.Chf, Ph.EtAc and Ph.Cr were also prominent with LC_50_ of 1.16, 6.35 and 0.93 mg/ml respectively. In anti-Pharaoh assay, again Ph.Sp and Ph.Chf were most potent fractions showing LC_50_ of < 0.01 mg/ml. Our current finding support previous insecticidal reports on saponins, and suggests that the saponins from *P. hydropiper* can be a cost-effective source of insecticidal compounds.

Other fractions with promising results can be subjected to activity guided isolation to obtain novel and more effective drugs against infectious diseases, insects and pests. We identified several insecticidal compounds in GC-MS analysis. Among these, the insecticidal activity of farnesol has also been reported [58]. Methyl palmitate and myristic acid also possess insecticidal properties [59, 60].

## Conclusions

Results of the current study indicate that *P. hydropiper* possess broad spectrum antimicrobial activity and signifies its potential as a source of therapeutic agent against bacterial and fungal infections. Further studies, regarding isolation and purification of novel bioactive component, can depict the precise potentials of the plant to restrain pathogenic microbes since the purified compounds may have even more efficacy with respect to inhibition of microbes. Our findings regarding antimicrobial and insecticidal activities, exhibited by extracts and saponins may offer scientific justification for the ethnomedicinal uses of the plant.

## Additional file

Additional file 1: Table S1. Result of GC, GC-MS analysis for the identification of compounds in Ph.Cr of *P.hydropiper. (DOCX 25 kb)*

## Abbreviations

*A. flavus*: *Aspergillus flavus*
*A. fumigatus*: *Aspergillus fumigatus*
*A. niger*: *Aspergillus niger*
*A. punctatum*: *Anobium punctatum*
CLSI: Clinical and laboratory standard institute
DIZ: Diameter of Inhibitory zone
*E. faecalis*: *Enterococcus faecalis*
*E.coli*: *Escherichia col*
*F. oxysporum*: *Fusarium oxysporum*
GC-MS: Gas chromatography-mass spectrometry
*K. pneumonia*: *Klebsella pneumonia*
*M. pharaonis*: *Monomorium pharaonis*
*MDR*: Multidrug resistant
MFCs: Minimum fungicidal concentration
MICs: Minimum inhibitory concentration
*P aeruginosa*: *Pseudomonas aeruginosa*
*P. hydropiper*: *Polygonum hydropiper*
*P. mirabilis*: *Proteus mirabilis*
Ph.Aq: Aqueous
Ph.Bt: *n*-Butanol
Ph.Chf: Chloroform
Ph.Cr: Crude extract
Ph.EtAc: Ethyl acetate
Ph.Hex: *n*-hexane
Ph.Sp: Crude saponins
*R. dominica*: *Rhyzopertha dominica*
*S. aureus*: *Staphylococcus aureu*
*S. typhi*: *Salmonella typhi*
SDA: Sabouraud dextrose Agar
*T. castaneum*: *Tribolium castaneum*
