# Supplementary material for: Chemical profiling, antimicrobial and insecticidal evaluations of Polygonum hydropiper L
Source: BMC Complement Altern Med. 2016 Dec 5;16:502. doi: 10.1186/s12906-016-1491-4 (PMC5139080; doi:10.1186/s12906-016-1491-4)
Supplement: Additional file 1: Table S1. — Result of GC, GC-MS analysis for the identification of compounds in Ph.Cr of P.hydropiper. (DOCX 25 kb) [file 12906_2016_1491_MOESM1_ESM.docx]

**Supplementary File/Additional File**

**Table S1:** Result of GC, GC-MS analysis for the identification of compounds in Ph.Cr of *P.hydropiper*.

| Compound Label | Common Name | RT | Formula | Hits (DB) |
| --- | --- | --- | --- | --- |
| Cpd 1:1,2,3-Propanetriol | Glycerol | 5.738 | C3H8O3 | 10 |
| Cpd 2: 3,4,5-Trimethyl-1-hexene | NF | 6.458 | C9H18 | 10 |
| Cpd 3:1-n-Heptene | Alpha heptylene | 6.584 | C7H14 | 10 |
| Cpd 4: 2,4(1H,3H)-Pyrimidinedione, 5-methyl | Thymin | 7.185 | C5H6N2O2 | 10 |
| Cpd 5: 3-Octanol, 2-methyl | NF | 7.563 | C9H20O | 10 |
| Cpd 7: 4-methyloxazole | NF | 7.563 | C4H5NO | 10 |
| Cpd 8: 2(S)-hydroxy-.gamma.-butyrolactone | NF | 7.985 | C4H6O3 | 5 |
| Cpd 9: 2,5-Pyrrolidinedione | Succinic imide | 8.228 | C4H5NO2 | 10 |
| Cpd 10: 4H-Pyran-4-one, 2,3-dihydro-3,5-dihydroxy-6-methyl- | NF | 8.726 | C6H8O4 | 10 |
| Cpd 11:Bicyclo[2.2.1]heptan-2-ol, 1,7,7-trimethyl-, endo | endo-Borneol | 9.341 | C10H18O | 10 |
| Cpd 12: 2-Octanol-2-D | 2-Octanol | 9.653 | C8H17DO | 1 |
| Cpd 13: 1,2-Benzenediol | Pyrocatechol | 9.878 | C6H6O2 | 10 |
| Cpd 14: Dihydro-3-methyl-4-acetoxy-2(3h)-furanone | NF | 10.022 | C7H10O4 | 10 |
| Cpd 15: Benzofuran, 2,3-dihydro | Coumaran | 10.342 | C8H8O | 10 |
| Cpd 16: 2-Furancarboxaldehyde, 5-(hydroxymethyl) | Oxymethylfurfurole | 10.522 | C6H6O3 | 10 |
| Cpd 17: 1H-Pyrrole-2,5-dione, 3-ethyl-4-methyl | Methylethylmaleimide | 10.665 | C7H9NO2 | 10 |
| Cpd 18: 2-Acetoxy-3-(benzyloxy)-1-isoprpoxypropane | NF | 10.809 | C15H22O4 | 1 |
| Cpd 19: Benzeneacetaldehyde | Hyacinthin | 10.95 | C8H8O | 10 |
| Cpd 20: 1-Benzyl-3,3,4-trimethylpiperidine | NF | 11.107 | C19H27NO4 | 9 |
| Cpd 21: 2-Methoxy-4-vinylphenol | p-Vinylguaiacol | 12.565 | C9H10O2 | 10 |
| Cpd 22: 1-(2,4,6-Trimethylphenyl)buta-1,3-diene | NF | 13.505 | C13H16 | 10 |
| Cpd 23: 2-Methylhepta-2,3-dien-1-ol | NF | 13.839 | C8H14O | 10 |
| Cpd 24: Diallyl(methyl)vinylsilane | Diallylvinylmethylsilane | 14.277 | C9H16Si | 10 |
| Cpd 25: Bicyclo[2.2.1]heptane-7-methanol, 2-hydroxy-1,7-dimethyl- |  | 15.25 | C10H18O2 | 10 |
| Cpd 26: .alpha.-Humulene | Humulene | 15.684 | C15H24 | 10 |
| Cpd 27: .beta.-D-Glucopyranose, 1,6-anhydro | Levoglucosan | 16.693 | C6H10O5 | 10 |
| Cpd 28: Benzoic acid, 4-hydroxy-3-methoxy-, methyl ester | Vanillic acid | 16.824 | C9H10O4 | 10 |
| Cpd 29: 2,6,6-trimethyl-2-hydroxycyclohexylidene acetolactone | NF | 17.225 | C11H16O2 | 10 |
| Cpd 30: Benzeneacetic acid, 4-hydroxy | 4-HPA | 17.409 | C8H8O3 | 10 |
| Cpd 31: Dodecanamide, N,N-bis(2-hydroxyethyl) | lauramide | 17.605 | C16H33NO3 | 10 |
| Cpd 32: Benzoic acid, 4-hydroxy-3-methoxy | Vanillic acid | 17.699 | C8H8O4 | 10 |
| Cpd 33: 2,6 - di - methoxy - 4 - vinyl - phenol | Vinyl syringol | 17.767 | C10H12O3 | 10 |
| Cpd 34: (-)-Caryophyllene oxide | Caryophyllene epoxide | 18.301 | C15H24O | 10 |
| Cpd 35: 2-Cyclopenten-1-one, 3-methyl-2-pentyl | Dihydrojasmone | 18.498 | C11H18O | 10 |
| Cpd 36:2,6,10-Dodecatrien-1-ol, 3,7,11-trimethyl | Dihydrofarnesol | 18.596 | C15H26O | 10 |
| Cpd 37: Humulene Oxide | Humulene Oxide | 18.806 | C15H24O | 10 |
| Cpd 38: Megastigmatrienone | Megastigmatrienone | 19.027 | C13H18O | 10 |
| Cpd 39: (-)-Caryophyllene oxide | B-Caryophyllene epoxide | 19.223 | C15H24O | 10 |
| Cpd 40: Tetracyclo[6.3.2.0(2,5).0(1,8)]tridecan-9-ol, 4,4-dimethyl- | NF | 19.297 | C15H24O | 10 |
| Cpd 41: 2H-Cyclopropa[g]benzofuran, 4,5,5a,6,6a,6b-hexahydro-4,4,6b-trimethyl-2- | NF | 19.36 | C15H22O | 10 |
| Cpd 42: (-)-Caryophyllene oxide | Caryophyllene epoxide | 19.681 | C15H24O | 10 |
| Cpd 43: (-)-Caryophyllene oxide | Caryophyllene epoxide | 19.937 | C15H24O | 10 |
| Cpd 44: 1,5-Cycloundecadiene, 9-(1-methylethylidene)- | NF | 20.133 | C14H22 | 10 |
| Cpd 45: Cyclohexane, 1-methyl-2,4-bis(1-methylethenyl)-, (1.alpha.,2.beta.,4.beta.)- | NF | 20.404 | C13H22 | 10 |
| Cpd 46: 2-Propenoic acid, 3-(4-hydroxyphenyl)-, methyl ester | Methyl p-coumarate | 21.411 | C10H10O3 | 10 |
| Cpd 47: n-Tetradecanoic acid | Myristic acid | 21.691 | C14H28O2 | 10 |
| Cpd 48: 1,3-Dihydro-1-ethoxy-1-methoxyisobenzofuran | NF | 21.821 | C11H14O3 | 3 |
| Cpd 49: Methyl-3-n-hexylbenzoate | Methyl 3-hexylbenzoate | 22.004 | C14H20O2 | 10 |
| Cpd 50: (-)-DRIM-7-EN-11-OL | Drimenol | 22.127 | C15H23O | 10 |
| Cpd 51: 1,7,7-Trimethyl-2-Oxa-Bicyclo(4.4.0)Dec-5-Ene | NF | 22.654 | C12H20O | 10 |
| Cpd 52: 2-Propenoic acid, 3-(4-hydroxyphenyl) | 4-Hydroxycinnamic acid | 22.792 | C9H8O3 | 10 |
| Cpd 53: Caryophyllene oxide | Caryophylene oxide | 23.31 | C15H24O | 10 |
| Cpd 54: 3,5-Octadiene, 4,5-diethyl-3,6-dimethyl- | NF | 23.909 | C14H26 | 10 |
| Cpd 55:7,11,15-TRIMETHYL,3-METHYLENE-1-HEXADECENE | Neophytadiene | 24.023 | C20H38 | 10 |
| Cpd 56: 2-Pentadecanone, 6,10,14-trimethyl | farnesyl acetone | 24.203 | C18H36O | 10 |
| Cpd 57: Velleral | Velleral | 24.657 | C15H20O2 | 10 |
| Cpd 58: 3,7,11,15-Tetramethyl-2-hexadecen-1-ol | NF | 24.833 | C20H40O | 10 |
| Cpd 59: 3-Hydroxy-4-methoxycinnamic acid | Cinnamic acid | 25.072 | C10H10O4 | 10 |
| Cpd 60: 3,7,11,15-Tetramethyl-2-hexadecen-1-ol | NF | 25.41 | C20H40O | 10 |
| Cpd 61: 1H-Indene, 2,3,3a,4-tetrahydro-3,3a,6-trimethyl-1-(1-methylethyl)- | NF | 25.534 | C15H24 | 10 |
| Cpd 62: Ethanone, 1-[2,3-dihydro-2-(1-methylethenyl)-5-benzofuranyl]-, (R) | NF | 26.411 | C13H14O2 | 6 |
| Cpd 63: 13-Docosenoic acid, methyl ester, (Z)- | Methyl erucate | 26.596 | C23H44O2 | 10 |
| Cpd 64: Hexadecanoic acid, methyl ester (CAS) | Methyl palmitate | 26.726 | C17H34O2 | 10 |
| Cpd 65: 1-Acetyl-3a,6-dimethyl-5,6-oxyoctahydroazulene | NF | 26.802 | C14H20O2 | 4 |
| Cpd 66: (-)-Campherenone | Campherenone | 27.179 | C15H24O | 10 |
| Cpd 67:(5a.alpha.,9a.beta.,9b.beta.)-5,5a,6,7,8,9,9a,9b-octahydro-6,6,9a trimethyln | NF | 27.471 | C15H22O2 | 10 |
| Cpd 68: Aristolene epoxide | Aristolene epoxide | 27.576 | C15H24O | 10 |
| Cpd 69: Hexadecanoic acid (CAS) | Palmitic acid | 27.912 | C16H32O2 | 10 |
| Cpd 70: (E)-3-Ethoxy-3-(p-tolyl)-2-propenal | NF | 28.067 | C12H14O2 | 1 |
| Cpd 71: 7,7-dichlorobicyclo[3.2.0]hept-2-en-6-one | NF | 29.445 | C15H24O | 10 |
| Cpd 72: 2H-2a,7-Methanoazuleno[5,6-b]oxirene, octahydro-3,6,6,7atetramethyl | NF | 29.577 | C15H24O | 10 |
| Cpd 73: Pent-3-ene-2-one, 3-phenyl-, oxime | NF | 29.869 | C11H13NO | 1 |
| Cpd 74: 2,6-Ditert-Butyl-4-Methylphenyl Phenylcarbamate | NF | 30.11 | C22H29NO2 | 10 |
| Cpd 75: 1,3,5-Cycloheptatriene, 3,4-diethyl-7,7-dimethyl- | NF | 30.221 | C13H20 | 10 |
| Cpd 76: 2,6,8-Trimethylbicyclo[4.2.0]oct-2-ene-1,8-diol | NF | 30.34 | C11H18O2 | 10 |
| Cpd 77: Iso-Velleral | Isovelleral | 30.565 | C15H20O2 | 10 |
| Cpd 78: 9,12-Octadecadienoic acid, methyl ester, (E,E)- | Methyl linolelaidate | 31.063 | C19H34O2 | 10 |
| Cpd 79: 11,14,17-Eicosatrienoic acid, methyl ester | NF | 31.215 | C21H36O2 | 10 |
| Cpd 80: 1-Dodecanol, 3,7,11-trimethyl- | Hexa-hydro-farnesol | 31.508 | C15H32O | 10 |
| Cpd 81: Heptadecanoic acid, 16-methyl-, methyl ester (CAS) | Methyl isostearate | 31.798 | C19H38O2 | 10 |
| Cpd 82: 5-Dodecyne | 5-C12H22 | 32.084 | C12H22 | 10 |
| Cpd 83: acrylic acid dodecanyl ester | Dodecyl acrylate | 32.216 | C15H28O2 | 10 |
| Cpd 84: (E and Z)-3-Ethylidenecyclohexene | Ethylidenecyclohexene | 32.242 | C8H12 | 10 |
| Cpd 85: 3-Methylbenzoic acid, 2-dimethylaminoethyl ester | NF | 32.425 | C12H17NO2 | 10 |
| Cpd 86: Octadecanoic acid | Vanicol | 32.613 | C18H36O2 | 10 |
| Cpd 87: Cedr-8(15)-en-9-ol (CAS) | Cedrenol | 32.694 | C15H24O | 10 |
| Cpd 88: Pyrrole-2,5-dione, 1-(2,5-dimethoxyphenyl)- | NF | 32.87 | C12H11NO4 | 1 |
| Cpd 89: Amide KK/Lauramide/ Dodecamide /Lauric amide | Dodecanamide | 32.948 | C12H25NO | 10 |
| Cpd 90:pentacyclo[9.1.0.0(2,4).0(5,7).0(8,10)]dodecane, 3,3,6,6,9,9,12,12-Octamethyl | NF | 33.028 | C20H32 | 10 |
| Cpd 91: 4-Methoxy-2,5-dihydrotoluene | dihydroanisole | 33.244 | C8H12O | 10 |
| Cpd 92: 6-Octenoic acid, 3-ethenyl-3,7-dimethyl-2-methylene-, methyl ester | NF | 33.395 | C14H22O2 | 2 |
| Cpd 93: 1-(3'-Methoxyprop-1'-ynyl)-2-methylcyclopropane | NF | 33.539 | C8H12O | 1 |
| Cpd 94: Bicyclo[3.2.1]oct-2-ene, 2-(phenylthio)- | NF | 33.63 | C14H16S | 1 |
| Cpd 95: 2-Cyclopenten-1-one, 2,3,4-trimethyl | NF | 33.868 | C8H12O | 6 |
| Cpd 96: Ledene oxide-(II) | Ledene oxide | 33.951 | C15H24O | 3 |
| Cpd 97: 3,6,8-Trimethyl-1-tetralone | NF | 34.58 | C13H16O | 10 |
| Cpd 98: 2,2,6-Trimethyl-1-(2-methyl-cyclobut-2-enyl)-hepta-4,6-dien-3-one | NF | 34.958 | C15H22O | 10 |
| Cpd 99: 2,2,6-Trimethyl-1-(3-methylbuta-1,3-dienyl)-7-oxabicyclo[4.1.0]heptan-3ol | NF | 35.453 | C14H22O2 | 10 |
| Cpd 100: Aristolenepoxide | Aristolenepoxide | 35.663 | C15H24O | 10 |
| Cpd 101: Eicosanoic acid, methyl ester (CAS) | Arachidic acid | 35.835 | C21H42O2 | 10 |
| Cpd 102: Z,Z,Z-1,4,6,9-Nonadecatetraene | Nonadecatetraene | 36.477 | C19H32 | 10 |
| Cpd 103: 6-Ethyl-8-iodo-2,3-dihydro-1H-quinolin-4-one | NF | 37.694 | C11H12INO | 2 |
| Cpd 104: 4-Acetyl-2-ethoxy-6-methoxyquinoline | NF | 38.475 | C14H15NO3 | 3 |
| Cpd 105: Undecane, 6-azido-6-pentyl- | Pentylmethyl Azide | 38.526 | C16H33N3 | 10 |
| Cpd 106: Eicosanoic acid, methyl ester | Arachidic acid | 39.295 | C21H42O2 | 10 |
| Cpd 107: 1,2-Benzenedicarboxylic acid, bis(2-ethylhexyl) ester | DEHP/DNOP | 39.616 | C24H38O4 | 10 |
| Cpd 108:6-Nonenamide, 8-methyl-N-vanillyl-, (E)- | Capsaicin | 39.791 | C18H27NO3 | 10 |
| Cpd 136: Dihydrocapsaicin | Dihydrocapsaicin | 40.137 | C18H29NO3 | 10 |
| Cpd 109: 3-(2-hydroxyethyl)-1-cyclohexene $$ 2-Cyclohexene-1-ethanol | NF | 41.493 | C8H14O | 1 |
| Cpd 110: Hexadecanoic acid, 15-methyl-, methyl ester | isoheptadecanoate | 42.374 | C18H36O2 | 10 |
| Cpd 111: Tricosane/n-Tricosane | n - tricosane | 44.757 | C23H48 | 10 |
| Cpd 112: Naphthalene-1-d, 2,3,4,4a,5,6,7,8-octahydro-4a-methyl | NF | 47.344 | C11H17D | 10 |
| Cpd 113: Cholesta-4,6-dien-3-ol, benzoate, (3.beta.) | NF | 47.823 | C34H48O2 | 10 |
| Cpd 114:4H-1-Benzopyran-4-one,3,5-dihydroxy-2(4-hydroxy-3methoxyphenyl)7 methoxy | NF | 48.714 | C17H14O7 | 10 |
| Cpd 115: dl-.alpha.-Tocopherol | Alpha Tocopherol | 48.991 | C29H50O2 | 10 |
| Cpd 116: 2,3-Dipropyl-4,6-diphenylpyridine | NF | 50.845 | C23H25N | 10 |
| Cpd 117: Pinoresinol | Pinoresinol | 50.958 | C20H22O6 | 1 |
| Cpd 118: Stigmast-5-en-3-ol, (3.beta.,24S) | Clionasterol | 53.741 | C29H50O | 3 |
| Cpd 119: Beta Bisabolene | Beta Bisabolene | 54.124 | C15H24 | 10 |
| Cpd 120: Benzene, (1-ethoxyethyl)pentafluoro |  | 57.107 | C10H9F5O | 1 |
| Cpd 121: 9H-Fluorene, 9-ethyl- | 9-Ethylfluorene | 57.744 | C15H14 | 3 |
| Cpd 122: component of Depo-Testadiol/Andro-Cyp | Testosterone cypionate | 58.028 | C27H40O3 | 4 |
| Cpd 123: 1,3 Dibenzoyl Propane | Dibenzoyl Propane | 58.839 | C17H16O2 | 10 |
| Cpd 124: 4-Chlorobutyric acid, pentadecyl ester | Chlorobutyric acid | 63.085 | C19H37ClO2 | 10 |

**NF:** Not found
